# Endozoochorous dispersal by herbivores and omnivores is mediated by germination conditions

**DOI:** 10.1186/s12898-020-00317-3

**Published:** 2020-08-31

**Authors:** Sorour Karimi, Mahmoud-Reza Hemami, Mostafa Tarkesh Esfahani, Christophe Baltzinger

**Affiliations:** 1 Department of Natural Resources, Isfahan University of Technology, 84156-83111 Isfahan, Iran; 2 INRAE Val de Loire, Research Unit Forest Ecosystems, Domaine des Barres, 45290 Nogent-sur-Vernisson, France; 3 Centre for Invasion Biology and Centre for Functional Biodiversity, School of Life Sciences, University of KwaZulu-Natal, Pietermaritzburg, 3209 South Africa

**Keywords:** Seed dispersal, Germination conditions, Plant-animal interactions, *Sus scrofa*, *Ursus arctos*, *Cervus elaphus*, *Capreolus capreolus*

## Abstract

**Background:**

Vertebrate-mediated seed dispersal is probably the main long distance dispersal mode. Through endozoochory, large mammals act as mobile links between habitats within and among forest patches. Along with other factors, their feeding regimes do affect their contribution as dispersal vectors. We conducted a cross-species comparative experiment involving two herbivores, red deer and roe deer; and two opportunistic omnivores, wild boar and brown bear, all occurring in the forest and steppe-forest ecotone habitats of the south-eastern Caspian region. We compared their role as endozoochorous seed dispersal agents by monitoring seedling emergence in their dungs under greenhouse and natural conditions.

**Results:**

In total, 3078 seedlings, corresponding to 136 plant taxa sprouted from 445 paired dung sub-samples, under greenhouse and natural conditions. Only 336 seedlings, corresponding to 36 plant taxa, emerged under natural conditions, among which five taxa did not appear under greenhouse conditions. Graminoids and forbs composed 91% of the seedlings in the greenhouse whereas shrubs were more abundant under natural conditions, representing 55% of the emerged seedlings. Under greenhouse conditions, first red deer and then wild boar dispersed more species than the other two mammals, while under natural conditions brown bear was the most effective vector. We observed remarkably higher species richness and seedling abundance per dung sub-sample under buffered greenhouse conditions than we did under natural conditions.

**Conclusions:**

The four sympatric mammals studied provided different seed dispersal services, both in terms of seedling abundance and species richness and may therefore be regarded as complementary. Our results highlight a positive bias when only considering germination under buffered greenhouse conditions. This must be taken into account when planning management options to benefit plant biodiversity based on the dispersal services concluded from greenhouse experiments.

## Background

The seed dispersal cycle “is a succession of processes whereby seeds produced by an adult plant are moved from the parent plant, germinate to seedlings, and recruit to adult plants, influencing the fruit and seed availability of the next generation” [1]. Seed dispersal also determines plant community dynamics and influences potential recruitment rates, recolonization, gene flow and consequently, genetic diversity [2]. Seed dispersal can also enable plant migration in response to environmental changes [3], accompany plant community responses to habitat fragmentation and also loss [4, 5], contribute to the soil seed bank [6], but is also responsible for spreading invasive exotic species [7].

Large herbivores are one of the most important drivers of vegetation dynamics in grazed ecosystems [8]. Through endozoochory, they act as mobile links between habitats within and among forest patches [9, 10]. In comparison with smaller herbivores, large herbivores consume more seeds, cause less damage to the seed during the chewing and ruminating processes [11] and disperse seeds over longer distances within their larger home ranges [12]. Herbivore traits such as body size, feeding regime and digestive physiology (i.e. ruminant or not) and spatio-temporal habitat preferences may affect the efficiency of endozoochorous dispersal [7, 13–15].

In addition to animal functional traits, plant phenology also matters. Seasonal variations in seed availability affect the number of species and seeds dispersed by the vectors [16]. Seed availability for herbaceous species peaks during spring and summer, while seeds from fleshy-fruited shrub species are more common in summer and early autumn in temperate zone.

Each step in the seed dispersal cycle is crucial [1] and this is also true for the establishment of seedlings emerging from faeces. Though several methods have been used to assess the composition, density and viability of seeds in dung content, most studies have investigated germination success under controlled greenhouse conditions or in standardised laboratory environments with a regular water supply and a relatively constant temperature [17, 18]. Such approaches actually indicate potential germination success rather than effective seed dispersal [19–21]. We therefore launched our study to test the effect of specific germination conditions on the outcome of endozoochorous plant dispersal.

We used a cross-species comparative experiment involving the four most common sympatric wild mammals occupying the forest and steppe-forest ecotone of the south-eastern Caspian region. There are two herbivores: an intermediate mixed feeder, the maral red deer (*Cervus elaphus maral*), and a browser, the roe deer (*Capreolus capreolus*) [22]; and two rather opportunistic omnivores (i.e. which make use of all available resources, including e.g. fruits, insects and earthworms) [23, 24]: the wild boar (*Sus scrofa*) and the brown bear (*Ursus arctos*).

We compared the plants germinating from their faeces by habitat and by season to investigate the different vectors’ contribution to the pool of plants dispersed. We formulated the following hypotheses:

1. Due to their differences in terms of feeding regime and selectivity, the different mammals should disperse different set of plants. The red deer as a mixed feeder should disperse more plants than the more selective roe deer. In addition, omnivores should disperse more fleshy-fruited plants than do herbivores.
2. The diversity of the plant species dispersed by the studied dispersal agents would vary temporally across the study area according to their various seed shedding periods and mammal habitat preferences. Seed dispersal by omnivores should peak in late summer/early autumn when fleshy fruits are abundant. By comparison, we expect abundant seed dispersal by herbivores in spring/early summer, when herbaceous plants predominate.
3. Due to variable abiotic conditions, in terms of water supply and temperature, we expect lower germination rates under natural conditions than under buffered greenhouse conditions.

## Results

### Seedling emergence under greenhouse conditions

From the total of 445 individual dung sub-samples, 129 plant species, from 29 families, germinated. Overall, 5.3% of the species could only be identified to the family level (seven *Poaceae* taxa) and 10% only to the genus level (13 taxa). Two seedlings died before they had grown sufficiently to enable identification. We did not observe any contaminating seedlings in the control pots (Additional file 1, Table 4).

More than 88% of the sub-samples contained germinated seeds: 97% for red deer, 91% for brown bear, 89% for wild boar and 88% for roe deer.

**Fig. 1:**
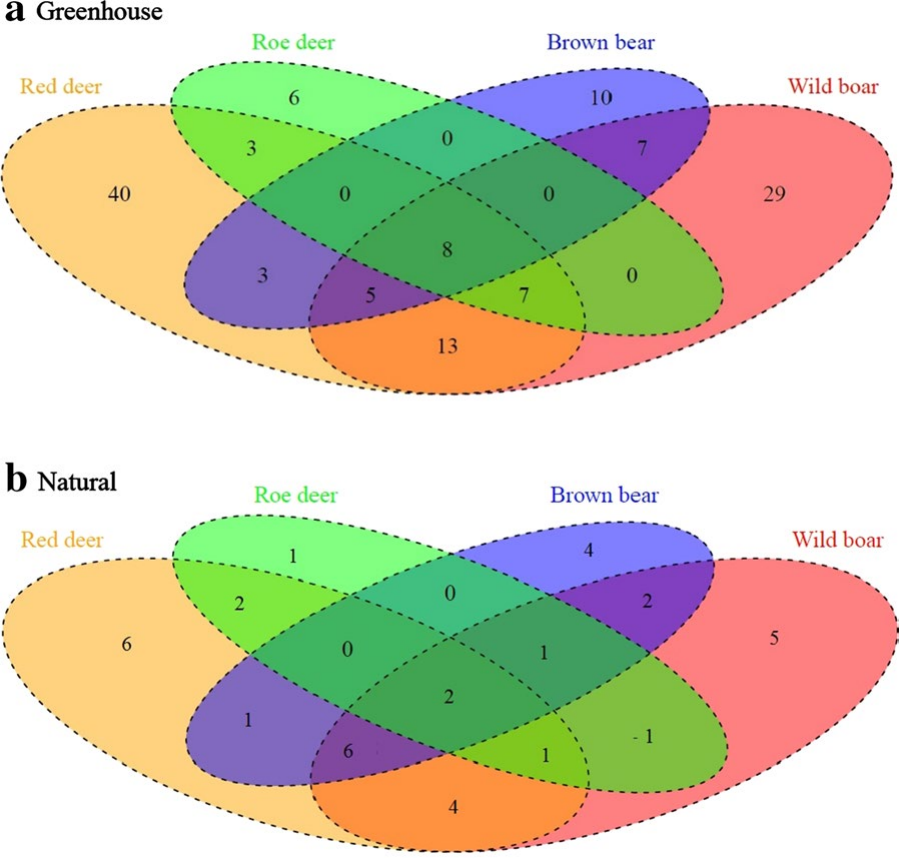
Number of species germinated by a given combination of vectors in the study area (both habitats combined), **a** under greenhouse conditions; and **b** under natural conditions

A greater number of seedlings generally meant a greater number of species (Spearman’s *rs *= 0.76; *P* < 0.0001), though some wild boar samples were dominated by a single species (i.e. *Urtica dioica*). Certain plant species were dispersed by a single animal vector: 40 by red deer, 29 by wild boar, ten by brown bear and six by roe deer (Additional file 1, Fig. S1a). Red deer dispersed the greatest number of plant species (Fig. 1a; Additional file 2; Table 4).

### Seedling emergence under natural conditions

By comparison, under natural conditions, fewer sub-samples provided seedlings: 55% for brown bear, 30% for wild boar, 26% for red deer, and 20% for roe deer. No seeds germinated from 67% of the dung sub-samples.

A total of 334 seedlings, corresponding to 36 plant species from 16 families germinated from the 445 paired dung sub-samples (Table 4; Additional file 1). In the control pots, we recorded the five following species: *Hesperis hyrcana*, *Lamium album*, *Torilis japonica*, *Nonea lutea*, and *Veronica persica*. These five species occurred more often in the control pots than in the non-control pots and were therefore excluded from further analyses.

Some plants were dispersed by a single vector (6 by red deer; 5 by wild boar; 4 by brown bear and 1 by roe deer) (Table 4; Fig. 1b).

### Seedling abundance and species richness

Based on the GLMM results for species richness, the best model included Animal species (Additional file 3). For seedling abundance, the best model included Animal species, Season and the interaction Animal × Season (Additional file 4).

Variability of seedling abundance per gram of faeces was low for all vectors, whether herbivores or omnivores, and whatever the season.

**Fig. 2:**
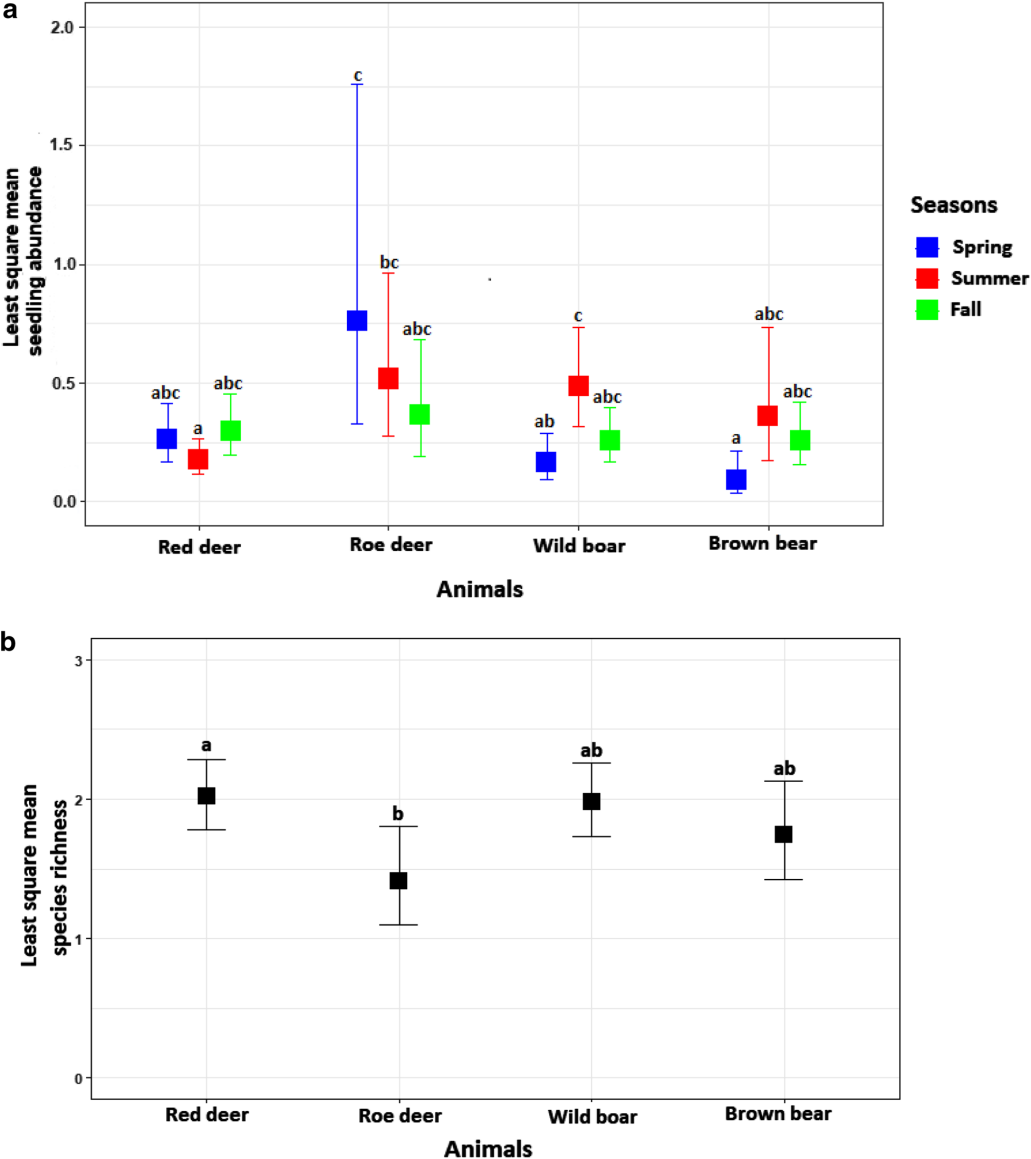
Plots of least square mean (with 95% confidence intervals) for (**a**) seedling abundance per gram of faeces vs. animal × season interaction, and (**b**) species richness per faeces vs. animal vector. Means sharing a letter are not significantly different (Turkey’s post hoc tests). The results were back-transformed to the original scale by taking antilogarithms of the least square means (LSM) and the 95% confidence intervals (CI)

Seedling abundance per gram of faeces was higher for roe deer than for omnivores in the spring. It was lower for red deer in the summer compared to wild boar and roe deer. Finally, we detected no differences in the fall (Fig. 2a, Table 1).

**Table 1:** The results of Tukey post hoc test for pairwise comparisons between dispersal vectors in terms of seedling abundance per gram of faeces in each season

	Brown bear	Wild boar	Red deer	Roe deer
Spring				
Brown bear		0.980	0.412	*0.008*
Wild boar	0.542 ± 0.504		0.890	*0.039*
Red deer	0.343 ± 0.300	0.632 ± 0.343		0. 340
Roe deer	0.118 ± 0.129	0.219 ± 0.194	0.346 ± 0.288	
Summer				
Brown bear		0.999	0.676	0.999
Wild boar	0.742 ± 0.515		*< 0.0001*	1.000
Red deer	2.046 ± 1.417	2.757 ± 0.962		*0.017*
Roe deer	0.695 ± 0.561	0.936 ± 0.555	0.340 ± 0.200	
Fall				
Brown bear		1.000	1.000	0.998
Wild boar	1.006 ± 0.484		0.999	0.994
Red deer	0.868 ± 0.414	0.862 ± 0.339		1.000
Roe deer	0.712 ± 0.482	0.707 ± 0.433	0.821 ± 0.486	

Values presented are the ratio ± 95% confidence interval (CI) in the lower triangle and the P‐value in the upper triangle. Significant differences are in italic

Species richness per faeces was higher for red deer than for roe deer; furthermore, it was similar for red deer, wild boar and brown bear (Fig. 2b, Table 2).

**Table 2:** The results of Tukey post hoc test for pairwise comparisons between dispersal vectors in terms of species richness per faeces

	Brown bear	Wild boar	Red deer	Roe deer
Brown bear		0.698	0.572	0.526
Wild boar	0.882 ± 0.200		0.993	0.060
Red deer	0.864 ± 0.192	0.980 ± 0.149		*0.034*
Roe deer	1.237 ± 0.378	1.402 ± 0.370	1.431 ± 0.370	

Values presented are the ratio ± 95% confidence interval (CI) in the lower triangle and the P‐value in the upper triangle. Significant differences are in italic

Habitat type (forest versus steppe-forest ecotone) had no effect on seedling abundance per gram of faeces or species richness per faeces, whatever the dispersal vector (Additional files 3 and 4).

### Natural versus greenhouse conditions

Of the total 136 plant taxa, 131 taxa appeared under greenhouse conditions versus only 36 taxa under natural conditions, five of which only germinated under natural conditions.

**Fig. 3:**
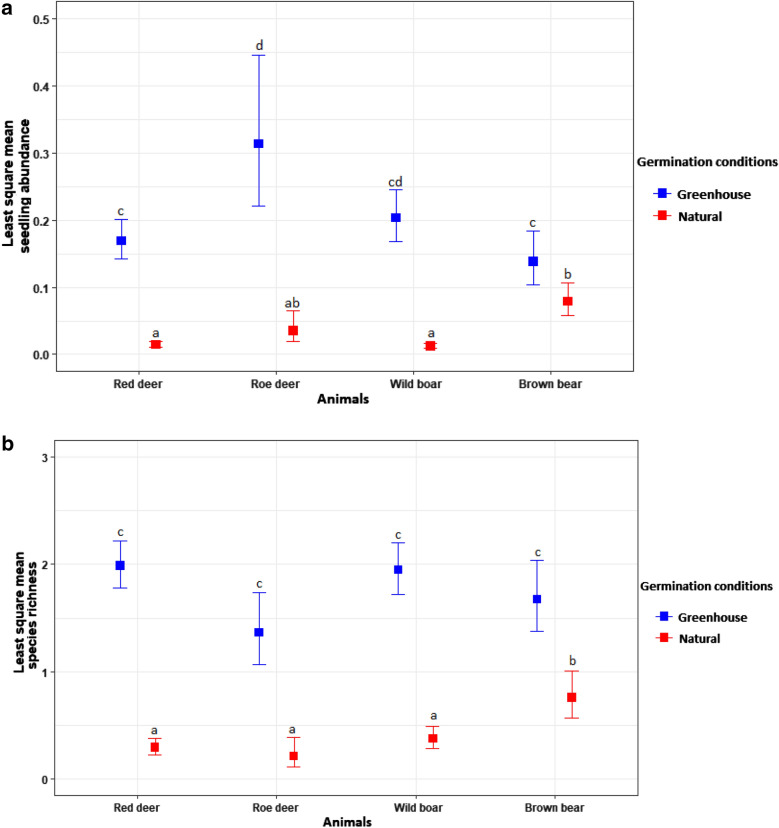
Plots of least square mean (with 95% confidence intervals) for (**a**) seedling abundance per gram of faeces vs. animal × germination condition interaction, and (**b**) species richness per faeces vs. animal × germination condition interaction. Means sharing a letter are not significantly different (Turkey’s post hoc tests). The results were back-transformed to the original scale by taking antilogarithms of the least square means (LSM) and the 95% confidence intervals (CI)

Based on the GLMM results for seedling abundance and species richness, the best model included Animal species, Germination conditions and the interaction Animal species × Germination conditions. Seedling abundance per gram of faeces and species richness per faeces sample were significantly higher under greenhouse than natural conditions (*P *≤ 0.001) for each animal species (Fig. 3).

The global lower number of species dispersed by each animal under natural conditions holds true also for each combination of vectors (Fig. 1), except for the set of species dispersed by red deer, wild boar and brown bear. Greenhouse and natural conditions’ sets contain different species. Half of the 6 species that germinated under natural conditions in red deer, wild boar and brown bear dungs produce fleshy fruits (drupes: *Sorbus torminalis* and *Rubus* sp. and berry *Berberis* sp.), whereas the other half produce dry fruits (achenes and caryopses).

We defined three groups of plants. The first group comprises species that germinated solely under greenhouse conditions. This group includes 62 herbs, 26 graminoids, six shrubs, one sub-shrub and one cushion plant (Additional file 1). The second group includes the five taxa (*Aegilops cylindrical*, *Cornus sanguinea*, *Calamintha nepeta*, *Rosa canina* and *Silene* sp.) that germinated only under natural conditions, though in low numbers. Finally, the third group includes the 31 plants that germinated under both natural and greenhouse conditions, though species generally (n = 21) emerged in lower abundance under natural conditions. However, the following eight taxa (*Cerasus* sp., *Parietaria officinalis*, *Poa masenderana*, *Poa nemoralis*, *Poa pratensis*, *Polygonum minus*, *Rumex sanguineus*. and *S. torminalis)* germinated more successfully under natural conditions.

Seedling abundance of the third plant group which germinated under both conditions was significantly higher under greenhouse conditions for roe deer (*U *= 7, *P *= 0.046) and wild boar (*U *= 34, *P *= 0.005), but not for red deer (*U *= 103.5, *P *= 0.184) or brown bear (*U *= 45.5, *P *= 0.382).

**Fig. 4:**
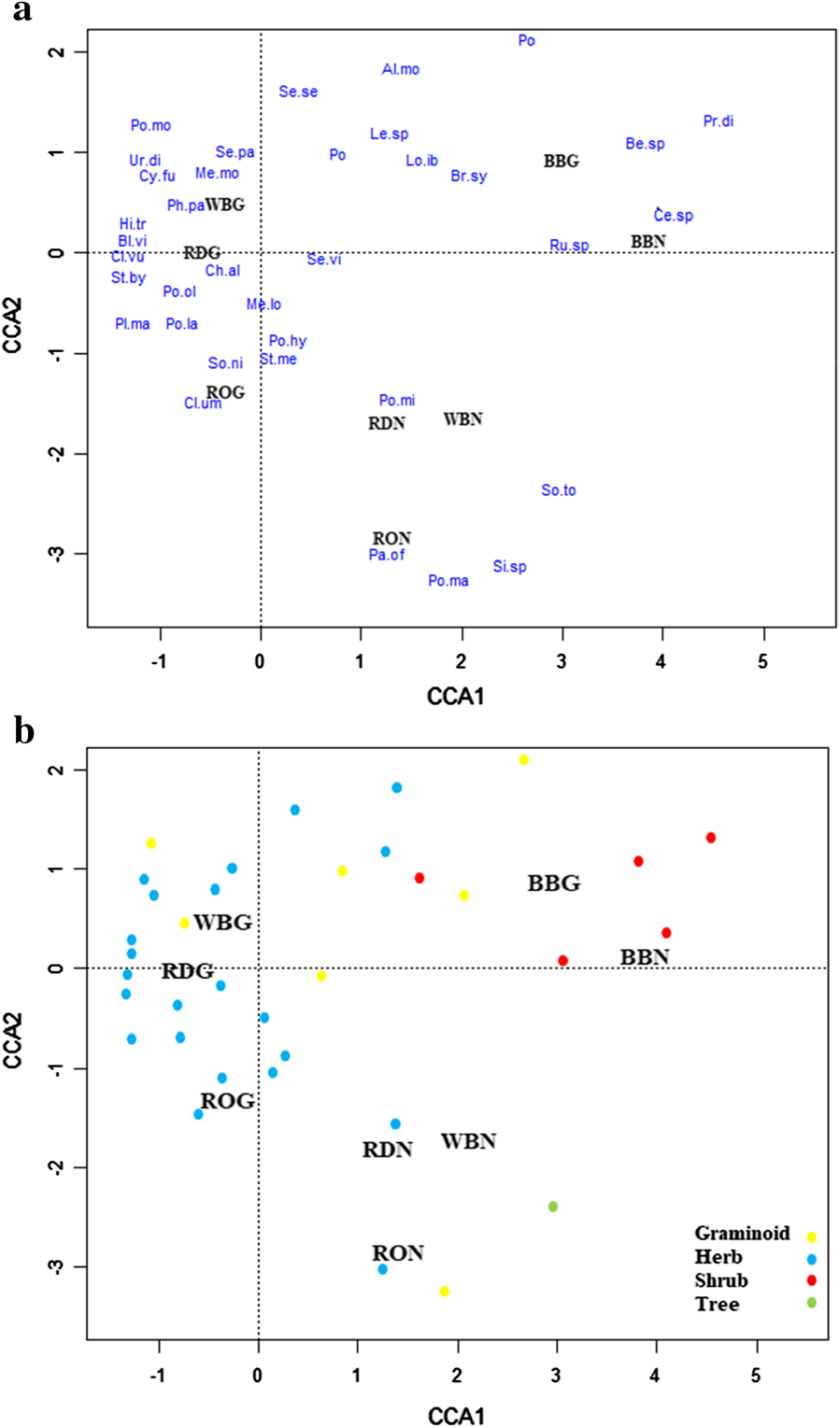
Biplots showing the results of the canonical correspondence analysis (CCA). The first plot (**a**) shows the position of each combined factor (animal plus germination condition, e.g. WBG = Wild Boar under Greenhouse conditions) on the first two CCA axes of the dispersed plant species. The second plot (**b**) shows the position of the same factors on the same axes of the dispersed species’ growth form (Graminoid, Herb, Shrub and Tree). Animal vectors: RO, Roe deer, RD, Red deer, BB, Brown bear, WB, Wild boar; and germination condition: G, Greenhouse conditions, N, Natural conditions. The plant species scientific names are written in shorthand form: the first two letters of the genus and the first two letters of the species (Additional file 5)

Dispersed plant species composition differed when animal and germination conditions were taken into account in the Canonical Correspondence Analysis (CCA) (*F*_*540, 7*_ = 2.534, *P *= 0.001; Fig. 4). Pairwise comparisons indicated strong differences in the composition of the dispersed species for each combination of animal and germination condition, except between red deer and roe deer (Table 3). Comparisons among animals also showed that red deer, roe deer and wild boar had greater differences with brown bear in terms of dispersed plant composition than they did with one another (Table 3). Comparisons among animals showed richer composition under greenhouse than under natural conditions for roe deer (*R *= 0.23; *P *= 0.001), red deer (R = 0.14; P = 0.001) and wild boar (R = 0.15; P = 0.001), but not for brown bear (R = 0.02; P = 0.091).

**Table 3:** ANOSIM results (r statistic) of pairwise tests for differences between animals using abundance data under greenhouse conditions (a) and natural conditions (b)

	Red deer	Roe deer	Brown bear
(a) Greenhouse conditions			
Roe deer	0.03		
Brown bear	0.11^***^	0.18^***^	
Wild boar	0.03^**^	0.09^***^	0.12^***^
(b) Natural conditions			
Roe deer	0.01		
Brown bear	0.10^***^	0.22^***^	
Wild boar	0.01^*^	0.05^**^	0.07^***^

*Significant differences: 0.001 ‘***’ 0.01 ‘**’ 0.05 ‘*’

## Discussion

The four animal vectors studied effectively dispersed a large set of the plants available in the Golestan National Park (GNP) through endozoochory. Several of the plant species that germinated frequently in our dung sub-samples or produced abundant seedlings (*U. dioica*, *Portulaca oleracea*, *Cyperus fuscus*, *Chenopodium album*, *Polygonum lapathifolium, Blitum virgatum*, *Berberis* sp. and *Stellaria media*) had been highlighted in previous studies [13, 15, 25, 26]. However, most of the species germinating in our study emerged only once and as a single seedling. This could be explained by accidental seed intake [7], forage contamination by surrounding seeds [27], low local abundance of the plant during the sampling time [28] or rare feeding bouts. However we should take into account that the five species that germinated only under natural conditions were also represented in low seedling numbers (i.e. 3 max.), four of them dispersed by red deer. As there were two typical fleshy fruited plants (*C. sanguinea* and *R. canina*), we cannot consider that the consumption was accidental.

Under greenhouse conditions, we found a higher proportion of dung sub-samples with emerging seedlings than previously reported for all our animal vectors except for brown bear [5, 15, 29], probably due to context-dependent plant local abundance and species richness. Nearly all the red deer dung sub-samples contained viable seeds. The species’ growth forms dispersed by red deer in our study are not so different from other studies, suggesting that our results most probably reveal better handling and germinating conditions than usual.

### Seedlings abundance and species richness

The differences among vectors in the seed content dispersed can be attributed to the animals’ dietary preferences (mixed feeders vs. browsers; [15, 30] and herbivores versus omnivores), digestive physiology (ruminant or not; [31]), body size, habitat preferences and ranging behaviour. These factors may also cause animal vectors to deposit the seeds in different habitats [7, 16].

Consistent with previous studies [7, 29], we found that grazers (i.e. red deer) effectively dispersed the highest number of species. We observed significant differences between the two deer species. The red deer has a wider diet than the roe deer and occupies a much larger home range [32]; these characteristics increase the animal’s encounter rate with numerous and diverse plant species and increases their consumption likelihood [13]. Apart from this difference between herbivores, however—and contrary to our expectations—the number of species dispersed did not differ among the other vectors, seasons, or between habitats. This can be attributed to the wide diversity of habitats and associated plants encountered in the GNP [33], making complementary food sources available in different seasons and across habitats. The similarity between omnivorous and herbivorous vectors in terms of number of species dispersed may also be due to an exceptionally high number of plants dispersed by the two omnivores in our study area, suggesting that they may have shifted their diet towards plant resources from animal sources [15, 30, 34]. Anthropogenic factors may also explain the absence of difference in species richness among our animal vectors. In the GNP, there has been a dramatic decline in large herbivores [35] mainly due to livestock grazing, habitat loss and intensive poaching [36]. The small remaining deer populations are now limited to the less disturbed sites, where, consequently, herbivory pressure has been increasing. In contrast, wild boar numbers have been increasing at the same time across a wide variety of habitats due to religious restrictions on eating wild boar meat [37], thus increasing the animal’s encounter rate with a wider range of plants. This corroborates a previous study [30] reporting more species dispersed by wild boar than by roe deer.

Even though the GNP hosts a very rich flora, some studies have reported higher numbers of plant species dispersed by vectors similar to brown bear [34]; by red and roe deer [7, 13], and by wild boar [15]. The preparation of the sub-samples in these studies could explain these results, as some plants have specific germination requirements other than cold stratification (e.g. warm stratification and exposure to smoke). A long germination period may have also increased the number of germinated seedlings and species [7] under both germination conditions. Finally, the difference in altitude (1616 m for the greenhouse and 450 m for the natural conditions) probably affected the germination pattern.

Only wild boar met our hypothesis on seed dispersal temporality with a peak in summer, whereas there were no significant patterns for the other three animal species. In general, seed density in dung depends on the feeding regime of the vectors and follows the seasonal pattern of plant seed-shedding [16].

As predicted, roe deer (but not red deer) in our study was more effective dispersal vectors than the omnivores in spring, when herbaceous plants are more frequent and abundant. However, the pattern was reversed in summer, wild boar dispersing more than red deer. Shrubs were mostly present in the omnivorous dung samples, collected in summer and autumn, when palatable fleshy fruits are available [34, 38].

In agreement with previous studies, roe deer dispersed more seeds per gram of faeces (i.e. seedling density) than wild boar [7, 26] in spring. There is no comparable study dealing with roe deer and brown bear. In summer, even red deer dispersed fewer viable seeds per gram of dung than did roe deer. During summer, roe deer probably selects more plants that produce seeds than red deer do. Red deer also feeds on graminoids in summer, but relative to what these animals consume, the portion represented by the seeds is higher for roe deer than for red deer, which needs more food quantity.

As a consequence of their feeding regime and body size [39], we also expected red deer to disperse more seeds than wild boar. However, the opportunistic feeding regime of wild boar considerably increased the number of seeds they dispersed (more than half of which emerged in summer). Indeed, in summer, wild boar dispersed higher seed density than did red deer. Some species like *Sonchus oleraceus* (75% of the seedlings emerged from roe deer dung in spring) and *P. lapathifolium* (accounting for one-third of the seedlings that emerged from red deer dung in autumn) make up the major part of seedling abundance in the specific season.

### Species composition

Both the functional traits of the animal vectors and plant phenology were reflected in the changes occurring in animal diet and habitat use across seasons, which in turn affected the composition of the plants dispersed. The largest dissimilarities among the sets of dispersed plant species were between brown bear and the three other vectors.

Most of the plant species dispersed in this study produced small seeds with no particular morphological structure or specific adaptations for endozoochory, with the exception of the seeds dispersed by the brown bear. This supports the “foliage is the fruit” hypothesis [27], which states that the edible vegetative parts of the plants act as the ecological ‘fruit’, or attractant reward. Brown bears preferentially selected fleshy fruits from shrubs and trees (*Berberis* sp., *Crataegus* sp. and *Cerasus* sp.), even though seeds from dry fruits were also dispersed by brown bear in lower densities than for fleshy fruits, and especially during spring. Indeed, though fleshy fruits from shrubs are an important source of energy during fall hyperphagia [40]; in spring, when those fruits are scarce, bears mainly rely on herbaceous plants and other vegetative parts (as well as animal carcasses) [34, 38].

Our results also reveal the potential of large native herbivores and omnivores, especially wild boar, to disperse ruderal, early-successional species, though only two of them (*P. oleracea* and *C. fuscus*) were dispersed in large quantities. A larger proportion of these species germinated from the wild boar dung collected during the dry season, when the animal spends more time feeding along roadsides. Wild boar can disperse some cultivated plants, like *Citrullus. vulgaris* and *Solanum. lycopersicum,* into natural areas when they take advantage of the food left by humans along roads or when they roam in agricultural areas [41]. Therefore overabundant wild boar populations may facilitate the spread of ruderal, early-successional or exotic plant species from such habitats, and consequently lead to mixed effects on species diversity [42]. On the other hand, decreasing deer populations translate into both reduced browsing pressure and reduced dispersal service with unpredictable outcomes for plant community composition. Therefore, conservation efforts should focus on the protection of animal populations which provide seed dispersal services to desired local plants [43].

#### Natural versus greenhouse germination conditions

As predicted, more plant species germinated under greenhouse than natural conditions, though the difference was low for brown bear. The greatest difference was observed for wild boar dung sub-samples, and may be linked to the large number of *U. dioica* seedlings, not observed under natural conditions.

From the 8 taxa that germinated more successfully under natural conditions only three really germinated in great number. *Cerasus* sp. and *S. torminalis*, both Rosaceae, germinated in higher number under natural conditions and these mainly concerned omnivores, whereas the third one *P. masendarana* was dispersed by the 4 vectors.

Different reasons can account for the lower seed germination rates found under natural conditions. First, artificial cold stratification was only applied to the dung sub-samples placed under greenhouse conditions whereas sub-samples under natural conditions depended on natural cold stratification. Most species must undergo a cold period to break seed dormancy and facilitate germination [44]. According to our results, buffered greenhouse conditions were probably beneficial to a highest proportion of species than were natural conditions. But as germination requirements are species dependent, some species, such as *C. sanguinea*, only germinated under more fluctuating natural conditions. Indeed, *C. sanguinea* seeds require a warm phase to degrade their stony endocarp [45] followed by chilling at 3 °C for 8–12 weeks to break seed dormancy [46]. For *R. canina*, 11 weeks of warm stratification followed by cold stratification appears to be the most appropriate treatment [47]. The regular water supply and relatively constant temperature under greenhouse conditions are likely to positively affect germination results if most of the plants require such conditions. *U. dioica*, for instance, has been frequently observed in dung [26, 48]; however, successful emergence under natural conditions did not occur in our study, probably because this plant prefers moist or damp soil [49]. Natural climatic fluctuations should facilitate germination for seeds with particular requirements [21]; we can therefore suppose that most of the plants dispersed in our study had quite similar needs. Finally, we did not check the seed content of the two sub-samples before submitting them to contrasting conditions [21]. This means that rare species, present as a single seed in the original dung sample would have germinated either in the greenhouse or under natural conditions, or not germinated at all, and this would lead to artificial heterogeneity between the sub-samples [21].

## Conclusions

We found that red deer dispersed more plant species, and that omnivores dispersed more fleshy fruited plants as expected. Concerning the seasonality of the dispersed plant species, only wild boar matched our prediction with higher seed dispersal during summer, whereas there were no significant seasonal differences for the other three animals. Our results mainly reveal that endozoochorous dispersal assessed under greenhouse conditions likely over-estimates establishment rate under natural conditions, and this is true for all animal species considered. Factors affecting germination success under natural conditions include the environmental filter (local abiotic conditions, dung decay speed) and biotic interactions among plants (competition, facilitation) or with other organisms (seed predation, herbivory, secondary seed dispersal) [50]. Nevertheless, the seeds that did not emerge from the dung under natural conditions over the course of this study may remain in the seed bank until abiotic edaphic conditions become suitable for germination. Extending studies similar to ours over the long term to cover the different climatic conditions in different years may provide more comprehensive results. In addition, to understand the ecological relevance of endozoochory, we need to mimic dung deposition in realistic conditions (i.e. compare germination in dung samples placed in different micro-habitats).

As revealed in our study, large mammals are important vectors for seed dispersal, and their loss or population reduction in natural ecosystems may have cascading effects on other taxa. We show that the four sympatric vectors we studied provide different, complementary seed dispersal services in terms of seasonality, seedlings and species number.

## Methods

### Study area

**Fig. 5:**
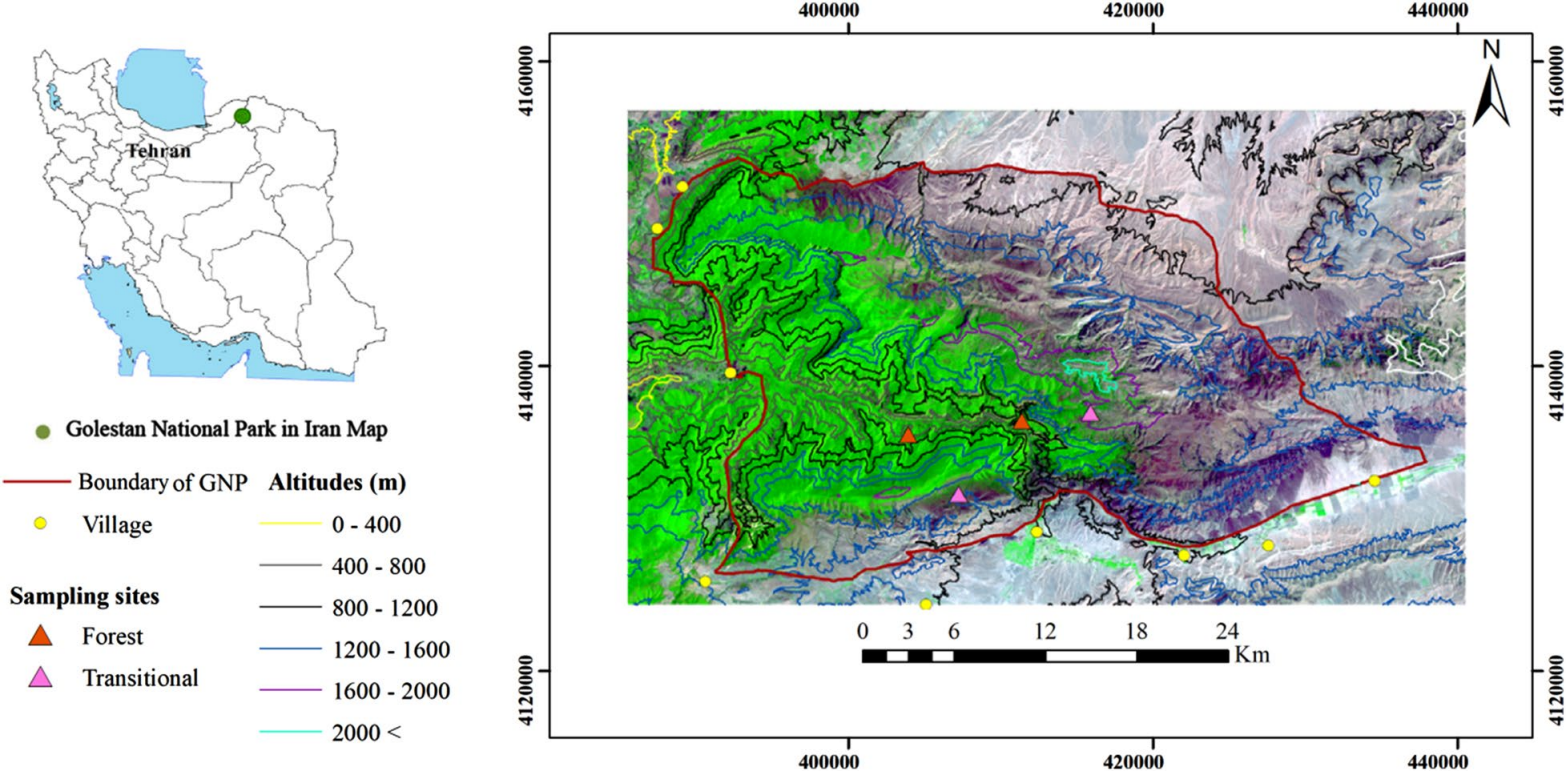
Location of Golestan National Park, highlighting the Hyrcanian forests (in green) in the western half and the surrounding steppes towards the east, north and south. Transitional vegetation zones occur in between and at high altitudes

GNP is situated in the north-eastern part of Iran (37°16’43′′ N 55°43′25′′ E-37°31′35′′ N 56°17′48′′ E) and is among the oldest and most diverse protected areas in the Middle East. It covers around 920 km^2^ of eastern Iranian Caspian forests with altitudes ranging from 450 to 2411 m above sea level (Fig. 5).

The average annual temperature varies from + 11.8 °C to + 18.8 °C. The climate is seasonal, marked by cold winters (January, mean temperature – 0.8 °C) and warm summers (July, mean temperature 23.3 °C). Summers with high temperatures in the dry regions can cause extremely hot, dry conditions in the east, south and northeast and a humid climate in the western part of the region [33]. Yearly precipitation ranges from 150 mm in the south-eastern part of the park to more than 1000 mm in the more central areas. The area receives 32.3%, 25.6%, 11.8%, and 30.3% of its annual rainfall during winter, spring, summer and fall, respectively.

The GNP lies across the Euro-Siberian and Irano-Turanian phytogeographical regions (Hyrcanian and Khorassan–Kopet–Dagh provinces, respectively). The park contains a wide range of flora and fauna, which are unique in many aspects. It encompasses diverse vegetation entities including Hyrcanian mesophytic forests, shrublands, scrublands (occasionally mixed with C4-composed grasslands), *Juniperus* sp., woodlands, mountain steppes and meadows, *Artemisia* sp. steppes, and communities composed of halophilous plants [33]. We divided these vegetation entities into two major habitat types where the target animal vectors are known to be present: Hyrcanian closed forests (hereafter, forests) and transitional scrub and Juniper woodlands (hereafter, steppe-forest ecotone). We therefore located our study plots within these two major habitat types, replicated twice; resulting in four sampling sites.

At the time of our study, there were about 257 (95% CI 91–423) red deer [51], 150 roe deer, 6000 (95% CI 3050–9906) wild boars [52], and 60 brown bears in the park (Annual population estimation by Golestan Provincial Department of Environment, 2016 unpublished data).

In our study area, the roe deer typically prefer a closed-forest habitat, which overlaps only slightly with the habitats favoured by the two omnivorous species. Red deer partly share the closed-forest and the steppe-forest ecotones with the other three species. The wild boar inhabit a wide range of habitats and brown bears usually prefer mountainous forested sites with high densities of fleshy-fruited shrubs and trees.

Home range (HR) sizes have not been evaluated in GNP for the four target animal species, however, other studies can provide information on the gradient of HR size among species (e.g. 17 ha, 81 ha, 283 ha and 5000 ha, respectively for roe deer, red deer, wild boar and brown bear [14, 53]).

### Dung collection and treatment

Dung samples were collected monthly from mid-May to November 2016 (spanning the seeding period) along random transects in the two habitat types. We could not find any faecal samples for brown bear or roe deer during certain months; therefore, samples were allocated to the following three seasons (spring, summer and autumn) to obtain at least two samples for each season-animal pair. We restricted dung collection to intact, fresh wet samples to limit post-dispersal modifications [7]. We prevented contamination from seeds sticking to the surface of the samples by removing the lowermost layer of the collected dung [15]. A small number of wild boar dung samples had been hollowed out by coprophagous beetles (5%) and were therefore discarded. The collected samples were air dried in paper bags for 10 days and weighed to the nearest 0.01 g. For red deer, wild boar and brown bear, we extracted two 20-g paired sub-samples from each faecal sample to investigate seedling emergence and plant establishment under greenhouse versus natural conditions. Because samples were lighter for roe deer (average weight of 5.67 ± 2.21 g; Table 4) than for the other three species, each individual roe deer dung sample was divided into two equally-sized sub-samples.

**Table 4:** Summary of the dispersed species assemblages by animal vector

	Brown bear		Wild boar		Roe deer		Red deer	
	G	N	G	N	G	N	G	N
Sample size (spring, summer, autumn)	64 (10, 11, 43)		149 (25,73,51)		50 (9, 20, 21)		182 (46, 71, 65)	
Mean weight of faeces (± SD)	202.81 ± 128.55		87.79 ± 49.85		5.67 ± 2.21		60.84 ± 19.09	
Total number of seedlings dispersed	268	153	1301	78	120	13	1053	90
Total number of species dispersed	33	16	69	22	24	8	79	22
Chao2 estimator of species dispersed (± SD)	43.7 ± 7.5	26.5 ± 10.1	107.0 ± 19.1	46.8 ± 24.0	28.6 ± 4.9	12 ± 4.7	115.1 ± 16.7	26.5 ± 4.2
Total number of genera dispersed	29	14	55	19	23	7	59	18
Total number of families dispersed	19	7	25	11	15	6	23	10
Top six plant taxa, in terms of total seedling abundance in faeces	*Berberis* sp.	*Berberis* sp.	*Urtica dioica*	*Cerasus* sp.	*Portulaca oleracea*	*Convolvulus arvensis*	*Blitum virgatum*	*Polygonum lapathifolium*
	*Rubus* sp.	*Cerasus* sp.	*Cyperus fuscus*	*Sorbus torminalis*	*Sonchus oleraceous*	*Stellaria media*	*Polygonum lapathifolium*	*Poa masenderana*
	*Crataegus* sp.	*Rubus* sp.	*Phleum paniculatum*	*Veronica beccabunga*	*Convolvulus arvensis*	*Poa nemoralis*	*Cyperus fuscus*	*Berberis* sp.
	*Cerasus* sp.	*Sorbus torminalis*	*Aegilops tauschii*	*Poa masenderana*	*Echinochloa crus*-*galli*	*Cornus sanguinea*	*Portulaca oleracea*	*Stellaria media*
	*Lonicera* sp.	*Rhamnus pallasii*	*Amaranthus blitoides*	*Rumex sanguineus*	*Apetiolata*	*Melilotus albus*	*Urtica dioica*	*Dysphania botrys*
	*Rhamnus pallasii*	*Prunus divaricata*	*Blitum virgatum*	*Berberis* sp.	*Phleum paniculatum*	*Poa masenderana*	*Amaranthus blitoides*	*Clinopodium umbrosum*

G, greenhouse conditions; N, natural conditions. Sample size was similar for greenhouse and natural conditions

### Germination experiments

Both the greenhouse and the natural experiments had a randomised block design with seven blocks (corresponding to sampling month) and four treatments (corresponding to animal vector). Over a 15-month period, we recorded the germinated seedling species weekly and then removed them. To obtain seedling species richness and abundance for each sampling season (spring, summer, autumn), we pooled the monthly data from May–June, July–September and October–November for each site and each animal vector.

### Greenhouse germination conditions

The samples were stored at 3–5 °C until field collections were completed [15], then each sample was carefully crushed to break apart the pellets. Each crushed sample was mixed with a similar volume of soil and sand and poured into pots (diameter 20 cm, depth 25 cm), making a layer approximately 1–2-cm thick. We then filled the pots with a 1:2:1 mixture (sand: soil: peat moss), which had previously been sterilised in an autoclave at 120 °C for 45 min [54].

The samples were then allowed to grow under natural daylight with daytime temperatures of around 25 °C in a greenhouse located at the Isfahan University of Technology (1616 m above sea level). The average minimum temperature was 18 °C. The samples were monitored every 2 days to maintain humidity. To prevent competition, we identified, counted, and removed the emerging seedlings as soon as possible. When no new seedlings emerged, the soil in each pot was thoroughly mixed and the experiment was continued for two more months to enable more deeply buried seeds to germinate [55]. To control for possible seed bank or seed rain contamination in the greenhouse, 30 control pots (without faecal samples) containing a similar substrate were placed among the pots with dung samples and were maintained under the same conditions.

### Natural germination conditions

To examine germination success under natural conditions, a 10 × 20 m exclosure was established (located in the Tangrah region: 37°23′53.7” N latitude, 55°47′54.4” E longitude, 450 m above sea level) and the experiment was carried out within the fenced area to prevent disturbance from grazing animals. To prevent any seeds in the soil seed bank from contaminating the experimental soil, we inverted the soil by bringing a layer of soil from a depth of more than 35 cm up to the surface of the experimental site [20]. Planting pots were filled with this deep soil and placed on the surface. The faecal samples were carefully crushed to break apart the pellets and were placed directly into each planting pot. To allow natural soil moisture into the planting pots and to improve rainwater drainage, the bottoms of the pots were removed. The faecal samples were not subjected to artificial cold treatment but were exposed to natural temperatures. Average annual rainfall was about 580 mm during the germination period. In order to control for air-borne seed input and soil seed bank content, seven control pots with soil only and no dung were positioned among the pots with dung samples for each month. Temperature and light were not controlled and no irrigation was applied during the experiment. The samples were completely exposed to natural climatic conditions. Emerged seedlings were identified to the species level whenever possible (11% could only be identified to the genus level).

### Data analysis

We built species accumulation curves with a Chao 2 estimator to assess how well we sampled the expected species richness [56]. These species accumulation curves helped us compare the different animal vectors under both greenhouse and natural conditions.

#### Greenhouse data analysis

We used generalized linear mixed models (GLMM) to compare seedling abundance and the number of species among dispersal vectors, season and habitat type (dung sample as the statistical unit). Negative binomial and Poisson regression models were respectively assigned for seedling abundance and species richness (count response variables) including additional over-dispersion in the model. Animal species (4 species), sampling season (spring, summer and autumn) and habitat (forest and steppe-forest ecotones) were fixed factors, and site within habitat was a random effect. The log-transformed weight of each dung sample was taken as an offset to account for differing sample weights.

We used the lsmeans package to obtain the predicted values for each combination of factors. We then performed a Tukey post hoc test for pairwise comparisons.

First, we fitted the full model to include all the main factors and Animal × Site and Animal × Season interactions (Additional files 6 and 7). The final model was obtained by backward stepwise selection. Best model selection was based on the lowest Akaike Information Criterion value (AIC).

#### Data analysis for natural versus greenhouse conditions

We used Poisson regression models to compare seedling abundance and species richness among animal species and between germination conditions (greenhouse vs. natural). First, we fitted the full model to include all the main factors and Animal species × Germination conditions interaction, with dung sample as the statistical unit. Best model selection was based on the lowest Akaike Information Criterion value (AIC). The lsmeans package and Tukey post hoc test were used to obtain the predicted values for each combination of factors and for pairwise comparisons, respectively.

Pairwise comparisons between greenhouse and natural conditions for seedling abundance of common plant species were made with the nonparametric Mann–Whitney U test.

We used canonical correspondence analysis (CCA) to compare the composition of germinating plants (square root of seedling abundance for each plant species) among the animal vectors and between germination conditions. Due to the high number of plant species, plotting priority was given to most abundant plant species in the dung samples, following Hill’s N2 diversity index. We used Monte-Carlo permutation tests (n = 999 permutations) to test the significance (*P* < 0.05) of the variables and the axes of the CCA. We compared differing plant species composition among animal vectors, and between germination conditions by an analysis of similarities (ANOSIM), with a Bray–Curtis similarity index ranging from zero (complete species overlap) to one (no species in common). This index excludes double-zero comparisons and does not weight rare or abundant species [57].

We performed all statistical analyses with the R 3.6.2. software (R Foundation for Statistical Computing, Vienna, AT) in the vegan 2.5–6 [58], venndiagram 1.6.2 [59], lme4 1.1–23 [60], lsmeans 2.30–0 [61] and MuMIn 1.43.17 [62] libraries.

## Supplementary information

**Additional file 1:** Alphabetical list of the plant species germinated from the faecal samples of the four different animal vectors. Fruit type (FT): Fleshy (+) or dry fruit (-), Life cycle (LF): A=Annual, B=Biennial, P=Perennial. Group: D=Dicotyledon, M=Monocotyledon. Degree of rarity in GNP (38): END=endangered, VUL=vulnerable, RAR=rare, NOT=non-threatened, UND=undetermined, UNK=unknown status. G=greenhouse conditions, N=natural conditions.

**Additional file 2:** Species accumulation curves for the plants dispersed by the different animal vectors (total dung mass for red deer: 3640 g; wild boar: 2980 g; brown bear: 1280 g; roe deer: 285.5 g) (a) for herbivores - top panel and (b) omnivores - bottom panel; under greenhouse (G) and natural (N) conditions, based on the Chao 2 estimator with 95% confidence intervals.

**Additional file 3:** Results from the best model selected by the Akaike Information Criterion for species richness per faeces.

**Additional file 4:** Results from the best model selected by the Akaike Information Criterion for seedling abundance per gram of faeces.

**Additional file 5:** The full scientific names of the plant species that are mentioned in the CCA plot.

**Additional file 6:** Estimated means ± SE obtained from generalized linear mixed effects models with a Poisson distribution with species richness per faeces as the response variable; animal, season and site, animal-site and animal-season interactions as fixed effects, and sample plot repetitions within each site as a random effect. One level of each factor (i.e. brown bear, ecotone, fall) is constrained in the “intercept” of the model.

**Additional file 7:** Estimated means ± SE obtained from generalized linear mixed effects models with a negative binomial distribution with seedling abundance per gram of faeces as the response variable; animal, season and site, animal-season and animal-site interactions as fixed effects; and sample plot repetitions within each site as a random effect. One level of each factor (i.e. brown bear, ecotone, fall) is constrained in the “intercept” of the model.

## Abbreviations

GNP: Golestan National Park
GLMM: Generalised linear mixed model
CCA: Canonical correspondence analysis
Chao2: Chao’s estimator of species richness

## Availability of data and materials

All data generated or analysed during this study accompanying with R scripts have been submitted to https://github.com/KARIMI66/Endozochorous-seed-dispersal-data.
