# Supplementary material for: Endozoochorous dispersal by herbivores and omnivores is mediated by germination conditions
Source: BMC Ecol. 2020 Aug 31;20:49. doi: 10.1186/s12898-020-00317-3 (PMC7457502; doi:10.1186/s12898-020-00317-3)
Supplement: Supplementary file 1 — Additional file 1: Alphabetical list of the plant species germinated from the faecal samples of the four different animal vectors. Fruit type (FT): Fleshy (+) or dry fruit (-), Life cycle (LF): A=Annual, B=Biennial, P=Perennial. Group: D=Dicotyledon, M=Monocotyledon. Degree of rarity in GNP (38): END=endangered, VUL=vulnerable, RAR=rare, NOT=non-threatened, UND=undetermined, UNK=unknown status. G=greenhouse conditions, N=natural conditions. [file 12898_2020_317_MOESM1_ESM.docx]

**Supplementary material**

# Endozoochorous dispersal by herbivores and omnivores depends on germination conditions

Sorour Karimi, Mahmoud-Reza Hemami, Mostafa Tarkesh Esfahani and Christophe Baltzinger

**Additional file 1** Alphabetical list of the plant species germinated from the faecal samples of the four different animal vectors. Fruit type (FT): Fleshy (+) or dry fruit (-), Life cycle (LF): A=Annual, B=Biennial, P=Perennial. Group: D=Dicotyledon, M=Monocotyledon. Degree of rarity in GNP (34): END=endangered, VUL=vulnerable, RAR=rare, NOT=non-threatened, UND=undetermined, UNK=unknown status. G=greenhouse conditions, N=natural conditions.

| **Species** | **Family** | **Growth form** | **FF** | **LF** | **Group** | **Degree of rarity** | **Brown bear** | | **Wild boar** | | **Red deer** | | **Roe deer** | | **Total number of seedlings dispersed** | |
| --- | --- | --- | --- | --- | --- | --- | --- | --- | --- | --- | --- | --- | --- | --- | --- | --- |
|  |  |  |  |  |  |  | **G** | **N** | **G** | **N** | **G** | **N** | **G** | **N** | **G** | **N** |
| *Aegilops cf cylindrica* | Poaceae | Graminoid | - | A | M | END |  |  |  |  |  | 1 |  |  |  | 1 |
| *Aegilops tauschii* | Poaceae | Graminoid | - | A | M | NOT |  |  | 37 |  | 15 |  |  |  | 52 |  |
| *Alliaria petiolata* | Brassicaceae | Herb | - | P | D | NOT |  |  |  |  |  |  | 6 |  | 6 |  |
| *Alyssopsis mollis* | Brassicaceae | Herb | - | B | D | NOT | 1 |  | 1 |  |  |  |  |  | 2 |  |
| *Alyssum tortuosum* | Brassicaceae | Non-spiny cushion form | - | P | D | NOT |  |  |  |  | 1 |  |  |  | 1 |  |
| *Amaranthus blitoides* | Chenopodiaceae | Herb | - | A | D | END |  |  | 35 |  | 48 |  |  |  | 83 |  |
| *Amaranthus cruentus* | Chenopodiaceae | Herb | - | A | D | END |  |  | 2 |  |  |  |  |  | 2 |  |
| *Arenaria serpyllifolia* | Caryophyllaceae | Herb | - | A | D | NOT |  |  | 3 |  | 18 |  |  |  | 21 |  |
| *Artemisia absinthium* | Asteraceae | Herb | - | P | D | RAR |  |  | 1 |  |  |  |  |  | 1 |  |
| Artemisia sp. | Asteraceae | Subshrub | - | P | D |  |  |  | 1 |  |  |  |  |  | 1 |  |
| *Atropa acuminate* | Solanaceae | Herb | + | P | D | END |  |  |  |  |  |  | 2 |  | 2 |  |
| *Berberis* sp. | Berberidaceae | Shrub | + | P | D |  | 100 | 53 | 8 | 5 |  | 9 |  |  | 108 | 67 |
| *Blitum virgatum* | Chenopodiaceae | Herb | + | A | D | VUL |  |  | 32 |  | 192 |  | 1 |  | 225 |  |
| *Brachypodium sylvaticum* | Poaceae | Graminoid | - | P | M | NOT |  |  |  |  | 5 |  |  |  | 5 |  |
| *Bromus briziformis* | Poaceae | Graminoid | - | A | M | RAR |  |  | 6 |  |  |  |  |  | 6 |  |
| *Bromus gedrosianus* | Poaceae | Graminoid | - | A | M | NOT |  | 1 | 1 |  |  |  |  |  | 1 | 1 |
| *Bromus madritensis* | Poaceae | Graminoid | - | A | M | SUN |  |  |  |  | 1 |  |  |  | 1 |  |
| *Bromus sterilis* | Poaceae | Graminoid | - | A | M | NOT |  |  |  |  | 3 |  |  |  | 3 |  |
| *Bromus tomentellus* | Poaceae | Graminoid | - | P | M | NOT |  |  | 3 |  |  |  |  |  | 3 |  |
| *Calamintha nepeta* | Lamiaceae | Herb | - | P | D | NOT |  |  |  |  |  | 2 |  |  |  | 2 |
| *Camelina rumelica* | Brassicaceae | Herb | - | A | D | NOT |  |  | 7 |  |  |  |  |  | 7 |  |
| *Capsella bursa-pastoris* | Brassicaceae | Herb | - | A | D | IND |  |  |  |  | 1 |  |  |  | 1 |  |
| *Cerasus* sp*.* | Rosaceae | Shrub | + | P | D |  | 16 | 36 | 3 | 10 |  |  |  |  | 19 | 46 |
| *Chenopodium album* | Chenopodiaceae | Herb | - | A | D | RAR | 2 |  | 24 | 4 | 12 | 2 | 3 |  | 41 | 6 |
| *Chenopodium vulvaria* | Chenopodiaceae. | Herb | - | A | D | END |  |  | 7 |  |  |  |  |  | 7 |  |
| *Cirsium osseticum* | Asteraceae | Thorny herb | - | P | D | VUL | 1 |  |  |  |  |  |  |  | 1 |  |
| *Citrullus vulgaris* | Cucurbitaceae | Herb | + | A | D | NOT |  |  | 3 |  |  |  |  |  | 3 |  |
| *Clinopodium umbrosum* | Lamiaceae | Herb | - | P | D | NOT |  |  | 3 | 1 | 17 | 4 | 4 |  | 24 | 5 |
| *Clinopodium vulgare* | Lamiaceae | Herb | - | P | D | NOT |  |  |  |  | 6 |  |  |  | 6 |  |
| *Colutea buhsei* | Fabaceae | Shrub | - | P | D | NOT |  |  |  |  | 2 |  |  |  | 2 |  |
| *Convolvulus arvensis* | Convolvulaceae | Herb | - | P | D | NOT | 2 |  | 8 |  | 13 | 3 | 12 | 3 | 35 | 6 |
| *Conyza Canadensis^§^* | Asteraceae | Herb | - | A | D | VUL |  |  | 2 | 1 |  |  |  |  | 2 | 1 |
| *Cornus sanguinea* | Cornaceae | Shrub | + | P | D | NOT |  | 2 |  | 1 |  |  |  | 2 |  | 5 |
| *Crataegus* sp. | Rosaceae | Shrub | + | P | D |  | 17 |  |  |  |  |  |  |  | 17 |  |
| *Crepis sancta* | Asteraceae | Herb | - | A | D | IND |  |  |  |  | 1 |  |  |  | 1 |  |
| *Cyperus fuscus* | Cyperaceae | Graminoid | - | A | M |  | 6 |  | 243 |  | 134 |  |  |  | 383 |  |
| *Draba huetii* | Brassicaceae | Herb | - | A | D | VUL |  |  | 4 |  | 14 |  |  |  | 18 |  |
| *Dysphania botrys* | Chenopodiaceae | Herb | - | A | D | END |  |  | 25 |  | 3 | 5 |  |  | 28 | 5 |
| *Echinochloa crus-galli* | Poaceae | Graminoid | - | A | M |  |  |  | 15 |  | 9 |  | 8 |  | 32 |  |
| *Elymus elongatus* | Poaceae | Graminoid | - | P | M |  |  |  | 1 |  |  |  |  |  | 1 |  |
| *Eragrostis minor* | Poaceae | Graminoid | - | A | M | END |  |  |  |  | 1 |  |  |  | 1 |  |
| *Erigeron acer* | Asteraceae | Herb | - | B | D | VUL |  |  | 1 |  |  |  |  |  | 1 |  |
| *Erodium oxyrrhynchum* | Geraniaceae | Herb | - | A | D | END |  |  |  |  | 6 |  |  |  | 6 |  |
| *Eruca sativa* | Brassicaceae | Herb | - | A | D | END | 2 |  |  |  |  |  |  |  | 2 |  |
| *Euphorbia bungei* | Euphorbiaceae | Herb | - | P | D | NOT |  |  |  |  | 2 |  |  |  | 2 |  |
| *Festuca valesiaca* | Poaceae | Graminoid | - | P | M | NOT |  |  | 9 |  |  |  |  |  | 9 |  |
| *Ficus carica* | Moraceae | Shrub | + | P | D | NOT |  |  | 12 |  |  |  |  |  | 12 |  |
| *Galium humifusum* | Rubiaceae | Herb | - | P | D | NOT |  |  |  |  | 7 |  |  |  | 7 |  |
| *Galium spurium* | Rubiaceae | Herb | - | A | D | IND | 3 |  |  |  |  |  |  |  | 3 |  |
| *Galium verum* | Rubiaceae | Herb | - | P | D | NOT |  |  |  |  |  |  | 1 |  | 1 |  |
| *Geranium kotschyi* | Geraniaceae | Herb | - | P | D | NOT |  |  |  |  | 1 |  |  |  | 1 |  |
| *Geranium rotundifolium* | Geraniaceae | Herb | - | A | D | END |  |  |  |  | 3 |  |  |  | 3 |  |
| *Heliotropium europaeum* | Boraginaceae | Herb | - | A | D | END |  |  | 2 |  | 1 |  | 1 |  | 4 |  |
| *Hibiscus trionum* | Malvaceae | Herb | - | A | D |  |  |  | 1 |  | 3 |  |  |  | 4 |  |
| *Holosteum umbellatum* | Caryophyllaceae | Herb | - | A | D |  |  |  | 1 |  |  |  |  |  | 1 |  |
| *Hordeum murinum* | Caryophyllaceae | Graminoid | - | A | M | END |  |  |  |  | 1 |  |  |  | 1 |  |
| *Juncus articulates* | Juncaceae | Graminoid | - | P | M | END |  |  |  |  | 6 |  |  |  | 6 |  |
| *Juncus inflexus* | Juncaceae | Graminoid | - | P | M | RAR |  |  | 2 |  |  |  |  |  | 2 |  |
| *Juncus* sp. | Juncaceae | Graminoid | - | P | M |  |  |  |  |  |  |  | 4 |  | 4 |  |
| *Lathyrus aphaca* | Fabaceae | Herb | - | A | D | RAR |  |  | 1 |  |  |  |  |  | 1 |  |
| *Lathyrus hirsutus* | Fabaceae | Herb | - | A | D | END |  |  |  |  | 3 |  |  |  | 3 |  |
| *Lepidium* sp. | Brassicaceae | Herb | - | A | D | END | 1 |  |  |  | 1 |  |  |  | 2 |  |
| *Lonicera floribunda* | Caprifoliaceae | Shrub | + | P | D | NOT |  |  |  |  | 1 |  |  |  | 1 |  |
| *Lonicera iberica* | Caprifoliaceae | Shrub | + | P | D | NOT | 12 |  | 1 |  | 9 | 1 |  |  | 22 | 1 |
| *Malcolmia Africana* | Brassicaceae | Herb | - | A | D | END |  |  | 15 |  | 1 |  | 2 |  | 18 |  |
| *Malva neglecta* | Malvaceae | Herb | - | P | D | END | 1 |  | 1 |  | 15 |  |  |  | 17 |  |
| *Malva sylvestris* | Malvaceae | Herb | - | P | D |  |  |  | 4 |  |  |  |  |  | 4 |  |
| *Medicago monantha* | Malvaceae | Herb | - | A | D | VUL |  |  | 3 |  | 4 |  |  |  | 7 |  |
| *Medicago monspeliaca* | Fabaceae | Herb | - | A | D | VUL | 2 |  | 5 |  | 7 |  |  |  | 14 |  |
| *Medicago sativa* | Fabaceae | Herb | - | P | D | NOT |  |  |  |  | 3 |  |  |  | 3 |  |
| *Melilotus albus* | Fabaceae | Herb | - | P | D | END |  |  | 5 |  | 2 |  | 5 | 1 | 12 | 1 |
| *Mentha longifolia* | Lamiaceae | Herb | - | P | D | RAR |  |  | 5 | 1 |  |  |  |  | 5 | 1 |
| *Mespilus germanica* | Rosaceae | Shrub | + | P | D | NOT | 9 |  |  |  |  |  |  |  | 9 |  |
| *Nepeta sintenisi* | Lamiaceae | Herb | - | P | D | RAR |  |  |  |  | 2 |  |  |  | 2 |  |
| *Onosma dichroantha* | Boraginaceae | Herb |  | P | D | NOT |  |  |  |  | 3 |  |  |  | 3 |  |
| *Parietaria officinalis* | Urticaceae | Herb | - | P | D | NOT |  |  |  | 2 | 1 |  |  |  | 1 | 2 |
| *Phleum paniculatum* | Poaceae | Graminoid | - | A | M | NOT | 3 | 1 | 45 | 1 | 22 |  | 5 |  | 75 | 2 |
| *Plantago lanceolata* | Plantaginaceae | Herb | - | P | D | END |  |  | 2 |  |  |  |  |  | 2 |  |
| *Plantago major* | Plantaginaceae | Herb | - | P | D | VUL |  |  |  |  | 6 |  | 1 |  | 7 |  |
| *Poa bulbosa* | Poaceae | Graminoid | - | P | M | NOT |  |  |  |  | 3 |  |  |  | 3 |  |
| *Poa densa* | Poaceae | Graminoid | - | P | M | NOT |  |  | 1 |  |  |  |  |  | 1 |  |
| *Poa masenderana* | Poaceae | Graminoid | - | A | M | NOT |  | 8 |  | 6 | 3 | 13 | 2 | 1 | 5 | 28 |
| *Poa nemoralis* | Poaceae | Graminoid | - | P | M | NOT |  |  |  |  | 1 | 1 |  | 2 | 1 | 3 |
| *Poa pratensis* | Poaceae | Graminoid | - | P | M | NOT |  |  |  | 5 | 1 | 4 |  |  | 1 | 9 |
| *Poa trivialis* | Poaceae | Graminoid | - | P | M | END |  |  |  |  | 6 |  |  |  | 6 |  |
| *Poaceae* | Poaceae | Graminoid | - | P | M |  | 6 |  | 8 |  | 6 |  |  |  | 20 |  |
| *Polygonum convolvulus* | Polygonaceae | Herb | - | A | D | NOT | 1 | 1 |  |  |  |  |  |  | 1 | 1 |
| *Polygonum hyrcanicum* | Polygonaceae | Herb | - | P | D | NOT | 1 | 1 | 9 | 4 | 8 | 2 | 1 |  | 19 | 7 |
| *Polygonum lapathifolium* | Polygonaceae | Herb | - | A | D |  |  |  | 14 | 2 | 162 | 19 | 1 |  | 177 | 21 |
| *Polygonum minus* | Polygonaceae | Herb | - | A | D | END |  | 1 | 3 | 3 |  | 1 |  |  | 3 | 5 |
| *Polypogon monspeliensis* | Poaceae | Graminoid | - | A | M |  |  |  | 13 |  |  |  |  |  | 13 |  |
| *Portulaca oleracea* | Portulacaceae | Herb | - | A | D | END | 9 |  | 27 |  | 88 |  | 27 |  | 151 |  |
| *Potentilla recta* | Rosaceae | Herb | - | P | D | NOT |  |  |  |  | 2 |  |  |  | 2 |  |
| *Prunella vulgaris* | Lamiaceae | Herb | - | P | D | NOT |  |  | 3 |  | 2 | 1 |  |  | 5 | 1 |
| *Prunus divaricata* | Rosaceae | Shrub | + | P | D | NOT | 8 | 8 |  |  |  |  |  |  | 8 | 8 |
| *Pulicaria dysenterica* | Asteraceae | Herb | - | P | D | END |  |  |  |  | 2 |  |  |  | 2 |  |
| *Rapistrum rugosum* | Brassicaceae | Herb | - | A | D | END |  |  | 1 |  |  |  |  |  | 1 |  |
| *Rhamnus pallasii* | Rhamnaceae | Shrub | + | P | D | NOT | 11 | 8 |  |  |  |  |  |  | 11 | 8 |
| *Rosa canina* | Rosaceae | Shrub | + | P | D | NOT |  |  |  | 1 |  |  |  |  |  | 1 |
| *Rubus* sp*.* | Rosaceae | Shrub | + | P | D |  | 30 | 19 | 4 | 3 |  | 2 |  |  | 34 | 24 |
| *Rumex sanguineus* | Polygonaceae | Herb | - | P | D |  |  | 1 |  | 6 | 1 | 3 |  | 1 | 1 | 11 |
| *Rumex tuberosus* | Polygonaceae | Herb | - | P | D | NOT |  |  |  |  | 1 |  |  |  | 1 |  |
| *Salvia atropatana* | Lamiaceae | Herb | - | P | D | NOT |  |  |  |  | 2 |  |  |  | 2 |  |
| *Salvia sclarea* | Lamiaceae | Herb | - | P | D | VUL |  |  |  |  | 1 |  |  |  | 1 |  |
| *Salvia virgata* | Lamiaceae | Herb | - | P | D |  |  |  | 1 |  |  |  |  |  | 1 |  |
| *Securigera securidaca* | Fabaceae | Herb | - | A | D | VUL | 1 |  | 3 |  |  |  |  |  | 4 |  |
| *Sedum pallidum* | Crassulaceae | Herb | - | A | D | IND | 1 |  | 3 |  | 2 |  |  |  | 6 |  |
| *Senecio glaucus* | Asteraceae | Herb | - | A | D | END |  |  |  |  | 1 |  |  |  | 1 |  |
| *Serratula latifolia* | Asteraceae | Herb | - | P | D | NOT |  |  |  |  | 1 |  |  |  | 1 |  |
| *Setaria viridis* | Poaceae | Graminoid | - | A | M | RAR | 9 | 1 | 6 | 1 | 14 | 2 | 4 |  | 33 | 4 |
| *Silene* sp. | Caryophyllaceae | Herb | - | A | D |  |  | 1 |  |  |  | 3 |  |  |  | 4 |
| *Sisymbrium irio* | Brassicaceae | Herb | - | A | D |  | 1 |  | 25 |  | 14 |  | 4 |  | 44 |  |
| *Solanum lycopersicum* | Solanaceae | Herb | + | A | D | NOT |  |  | 2 |  |  |  |  |  | 2 |  |
| *Solanum nigrum* | Solanaceae | Herb | + | A | D | END |  |  | 1 |  | 1 | 1 |  |  | 2 | 1 |
|  |  |  |  |  |  |  |  |  |  |  |  |  |  |  |  |  |
| *Sonchus asper* | Asteraceae | Herb | - | A | D | END |  |  | 6 |  | 4 |  |  |  | 10 |  |
| *Sonchus oleraceous* | Asteraceae | Herb | - | A | D | END |  |  |  |  |  |  | 20 |  | 20 |  |
| *Sorbus torminalis* | Rosaceae | Tree | + | P | D | NOT | 2 | 11 |  | 10 | 2 | 4 |  |  | 4 | 25 |
| *Stachys*  *byzantina* | Lamiaceae | Herb | - | P | D | NOT |  |  |  |  | 20 |  | 1 |  | 21 |  |
| *Stellaria holostea* | Caryophyllaceae | Herb | - | P | D | NOT |  |  | 1 |  |  |  |  |  | 1 |  |
| *Stellaria media* | Caryophyllaceae | Herb | - | A | D | RAR | 5 |  | 10 | 1 | 15 | 7 | 4 | 2 | 34 | 10 |
| *Tanacetum parthenium* | Poaceae | Herb | - | P | D | NOT |  |  |  |  | 1 |  |  |  | 1 |  |
| *Thlaspi perfoliatum* | Brassicaceae | Herb | - | A | D | NOT |  |  |  |  | 2 |  |  |  | 2 |  |
| *Trifolium campestre* | Fabaceae | Herb | - | A | D | VUL |  |  |  |  | 9 |  |  |  | 9 |  |
| *Urtica dioica* | Urticaceae | Herb | - | P | D | NOT |  |  | 550 |  | 73 |  |  |  | 623 |  |
| *Valerianella* sp. | Valerianaceae | Herb | - | A | D |  | 1 |  |  |  |  |  |  |  | 1 |  |
| *Veronica beccabunga* | Scrophulariaceae | Herb | - | P | D | END | 3 |  | 19 | 7 |  |  |  |  | 22 | 7 |
| *Veronica hederifolia* | Scrophulariaceae | Herb | - | A | D | VUL | 1 |  |  |  |  |  |  |  | 1 |  |
| *Vulpia persica* | Poaceae | Graminoid | - | A | M | END |  |  |  | 3 |  |  | 1 | 1 | 1 | 4 |
