# Supplementary material for: Endozoochorous dispersal by herbivores and omnivores is mediated by germination conditions
Source: BMC Ecol. 2020 Aug 31;20:49. doi: 10.1186/s12898-020-00317-3 (PMC7457502; doi:10.1186/s12898-020-00317-3)
Supplement: Supplementary file 2 — Additional file 2: Species accumulation curves for the plants dispersed by the different animal vectors (total dung mass for red deer: 3640 g; wild boar: 2980 g; brown bear: 1280 g; roe deer: 285.5 g) (a) for herbivores - top panel and (b) omnivores - bottom panel; under greenhouse (G) and natural (N) conditions, based on the Chao 2 estimator with 95% confidence intervals. [file 12898_2020_317_MOESM2_ESM.docx]

**Supplementary material**

# Endozoochorous dispersal by herbivores and omnivores depends on germination conditions

Sorour Karimi, Mahmoud-Reza Hemami, Mostafa Tarkesh Esfahani and Christophe Baltzinger


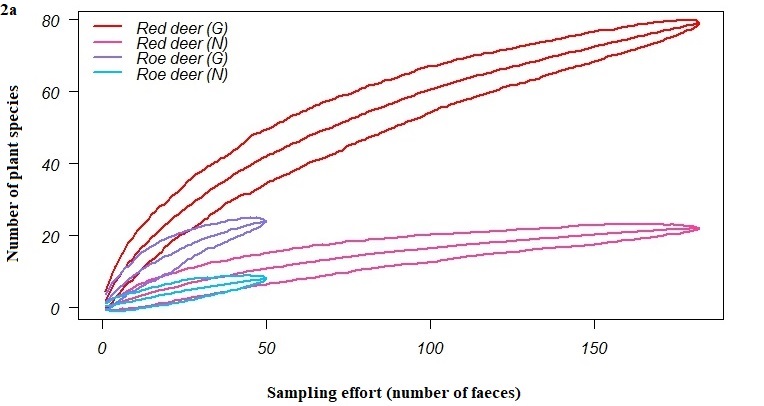


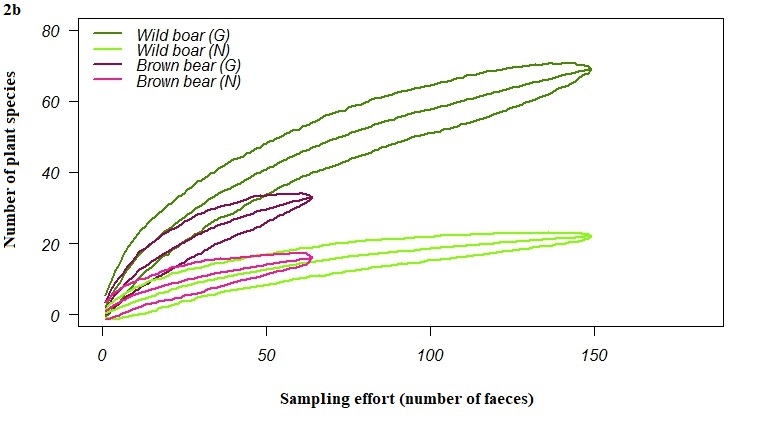


**Additional file 2** Species accumulation curves for the plants dispersed by the different animal vectors (total dung mass for red deer: 3640 g; wild boar: 2980 g; brown bear: 1280 g; roe deer: 285.5 g) (a) for herbivores - top panel and (b) omnivores - bottom panel; under greenhouse (G) and natural (N) conditions, based on the Chao 2 estimator with 95% confidence intervals.
