# Supplementary material for: Endozoochorous dispersal by herbivores and omnivores is mediated by germination conditions
Source: BMC Ecol. 2020 Aug 31;20:49. doi: 10.1186/s12898-020-00317-3 (PMC7457502; doi:10.1186/s12898-020-00317-3)
Supplement: Supplementary file 3 — Additional file 3: Results from the best model selected by the Akaike Information Criterion for species richness per faeces. [file 12898_2020_317_MOESM3_ESM.docx]

**Supplementary material**

# Endozoochorous dispersal by herbivores and omnivores depends on germination conditions

Sorour Karimi, Mahmoud-Reza Hemami, Mostafa Tarkesh Esfahani and Christophe Baltzinger

| **Additional file 3** Results from the best model selected by the Akaike Information Criterion for species richness per faeces | | | | | | | | | | |
| --- | --- | --- | --- | --- | --- | --- | --- | --- | --- | --- |
| Intercept | Animal | Season | Habitat | Animal: Season | Animal: Habitat | Df | LogLik | AICc | Delta | Weight |
| 0.556 | + |  |  |  |  | 5 | -711.309 | 1432.800 | 0 | 0.356 |
| 0.545 | + | + |  |  |  | 7 | -709.879 | 1434.000 | 1.260 | 0.190 |
| 0.551 | + |  | + |  |  | 6 | -711.302 | 1434.800 | 2.040 | 0.128 |
