# Supplementary material for: Endozoochorous dispersal by herbivores and omnivores is mediated by germination conditions
Source: BMC Ecol. 2020 Aug 31;20:49. doi: 10.1186/s12898-020-00317-3 (PMC7457502; doi:10.1186/s12898-020-00317-3)
Supplement: Supplementary file 4 — Additional file 4: Results from the best model selected by the Akaike Information Criterion for seedling abundance per gram of faeces. [file 12898_2020_317_MOESM4_ESM.docx]

**Supplementary material**

# Endozoochorous dispersal by herbivores and omnivores depends on germination conditions

Sorour Karimi, Mahmoud-Reza Hemami, Mostafa Tarkesh Esfahani and Christophe Baltzinger

| **Additional file 4** Results from the best model selected by the Akaike Information Criterion for seedling abundance per gram of faeces | | | | | | | | | | |
| --- | --- | --- | --- | --- | --- | --- | --- | --- | --- | --- |
| Intercept | Animal | Season | Habitat | Animal: Season | Animal: Habitat | Df | LogLik | AICc | Delta | Weight |
| -1.350 | + | + |  | + |  | 14 | -1228.589 | 2486.200 | 0.000 | 0.669 |
| -1.331 | + | + | + | + |  | 15 | -1228.581 | 2488.300 | 2.130 | 0.231 |
| -1.320 | + | + | + | + | + | 18 | -1226.172 | 2489.900 | 3.800 | 0.100 |
