# Supplementary material for: Endozoochorous dispersal by herbivores and omnivores is mediated by germination conditions
Source: BMC Ecol. 2020 Aug 31;20:49. doi: 10.1186/s12898-020-00317-3 (PMC7457502; doi:10.1186/s12898-020-00317-3)
Supplement: Supplementary file 5 — Additional file 5: The full scientific names of the plant species that are mentioned in the CCA plot. [file 12898_2020_317_MOESM5_ESM.docx]

**Supplementary material**

# Endozoochorous dispersal by herbivores and omnivores depends on germination conditions

Sorour Karimi, Mahmoud-Reza Hemami, Mostafa Tarkesh Esfahani and Christophe Baltzinger

| **Additional file 5** The full scientific names of the plant species that are mentioned in the CCA plot | |
| --- | --- |
| Abbreviation name | Full scientific name |
| Al.mo | *Alyssopsis mollis* |
| Be.sp | *Berberis* sp. |
| Bl.vi | *Blitum virgatum* |
| Br.sy | *Brachypodium sylvaticum* |
| Ce.sp | *Cerasus* sp. |
| Ch.al | *Chenopodium album* |
| Cl.um | *Clinopodium umbrosum* |
| Cl.vu | *Clinopodium vulgare* |
| Cy.fu | *Cyperus fuscus* |
| Hi.tr | *Hibiscus trionum* |
| Le.ib | *Lonicera iberica* |
| Le.sp | *Lepidium* sp. |
| Me.lo | *Mentha longifolia* |
| Me.mo | *Medicago monantha* |
| Pa.of | *Parietaria officinalis* |
| Ph.pa | *Phleum paniculatum* |
| Pl.ma | *Plantago major* |
| Po | Poaceae |
| Po.hy | *Polygonum hyrcanicum* |
| Po.la | *Polygonum lapathifolium* |
| Po.ma | *Poa masenderana* |
| Po.mi | *Polygonum minus* |
| Po.mo | *Polypogon monspeliensis* |
| Po.ol | *Portulaca oleracea* |
| Pr.di | *Prunus divaricata* |
| Ru.sp | *Rubus* sp*.* |
| Se.pa | *Sedum pallidum* |
| Se.se | *Securigera securidaca* |
| Se.vi | *Setaria viridis* |
| Si.sp | *Silen* sp. |
| So.ni | *Solanum nigrum* |
| So.to | *Sorbus torminalis* |
| St.by | *Stachys*  *byzantina* |
| St.me | *Stellaria media* |
| Ur.di | *Urtica dioica* |
