# Supplementary material for: Endozoochorous dispersal by herbivores and omnivores is mediated by germination conditions
Source: BMC Ecol. 2020 Aug 31;20:49. doi: 10.1186/s12898-020-00317-3 (PMC7457502; doi:10.1186/s12898-020-00317-3)
Supplement: Supplementary file 6 — Additional file 6: Estimated means ± SE obtained from generalized linear mixed effects models with a Poisson distribution with species richness per faeces as the response variable; animal, season and site, animal-site and animal-season interactions as fixed effects, and sample plot repetitions within each site as a random effect. One level of each factor (i.e. brown bear, ecotone, fall) is constrained in the “intercept” of the model. [file 12898_2020_317_MOESM6_ESM.docx]

**Supplementary material**

# Endozoochorous dispersal by herbivores and omnivores depends on germination conditions

Sorour Karimi, Mahmoud-Reza Hemami, Mostafa Tarkesh Esfahani and Christophe Baltzinger

**Additional file 6** Estimated means ± SE obtained from generalized linear mixed effects models with a Poisson distribution with species richness per faeces as the response variable; animal, season and site, animal-site and animal-season interactions as fixed effects, and sample plot repetitions within each site as a random effect. One level of each factor (i.e. brown bear, ecotone, fall) is constrained in the "intercept" of the model.

| Fixed effects | Estimate | Std. Error | z value | Pr(>\|z\|) |
| --- | --- | --- | --- | --- |
| Intercept | 0.569 | 0.135 | 4.230 | 0.233e-04^***^ |
| Animal (Red deer) | 0.164 | 0.169 | 0.969 | 0.333 |
| Animal (Roe deer) | -1.489 | 1.047 | -1.422 | 0.155 |
| Animal (Wild boar) | 0.007 | 0.177 | 0.041 | 0.967 |
| Site (Forest) | 0.022 | 0.231 | 0.094 | 0.925 |
| Season (Spring) | -0.209 | 0.320 | -0.652 | 0.514 |
| Season (Summer) | 0.017 | 0.274 | 0.063 | 0.950 |
| Animal (Red deer): Site (Forest) | -0.133 | 0.252 | -0.528 | 0.597 |
| Animal (Roe deer): Site (Forest) | 1.204 | 1.045 | 1.152 | 0.249 |
| Animal (Wild boar): Site (Forest) | 0.096 | 0.254 | 0.378 | 0.706 |
| Animal (Red deer): Season (Spring) | 0.193 | 0.348 | 0.555 | 0.579 |
| Animal (Roe deer): Season (Spring) | 0.140 | 0.475 | 0.295 | 0.768 |
| Animal (Wild boar): Season (Spring) | 0.135 | 0.368 | 0.366 | 0.715 |
| Animal (Red deer): Season (Summer) | 0.078 | 0.299 | 0.262 | 0.793 |
| Animal (Roe deer): Season (Summer) | 0.185 | 0.383 | 0.482 | 0.630 |
| Animal (Wild boar): Season (Summer) | 0.095 | 0.303 | 0.315 | 0.753 |

Significant codes: 0 ‘^***^’ 0.001 ‘^**^’ 0.01 ‘*’ 0.05 ^‘.’^
