# Supplementary material for: Endozoochorous dispersal by herbivores and omnivores is mediated by germination conditions
Source: BMC Ecol. 2020 Aug 31;20:49. doi: 10.1186/s12898-020-00317-3 (PMC7457502; doi:10.1186/s12898-020-00317-3)
Supplement: Supplementary file 7 — Additional file 7: Estimated means ± SE obtained from generalized linear mixed effects models with a negative binomial distribution with seedling abundance per gram of faeces as the response variable; animal, season and site, animal-season and animal-site interactions as fixed effects; and sample plot repetitions within each site as a random effect. One level of each factor (i.e. brown bear, ecotone, fall) is constrained in the “intercept” of the model. [file 12898_2020_317_MOESM7_ESM.docx]

**Supplementary material**

# Endozoochorous dispersal by herbivores and omnivores depends on germination conditions

Sorour Karimi, Mahmoud-Reza Hemami, Mostafa Tarkesh Esfahani and Christophe Baltzinger

**Additional file 7** Estimated means ± SE obtained from generalized linear mixed effects models with a negative binomial distribution with seedling abundance per gram of faeces as the response variable; animal, season and site, animal-season and animal-site interactions as fixed effects; and sample plot repetitions within each site as a random effect. One level of each factor (i.e. brown bear, ecotone, fall) is constrained in the "intercept" of the model.

| Fixed effects | Estimate | Std. Error | z value | Pr(>\|z\|) |
| --- | --- | --- | --- | --- |
| Intercept | -1.319 | 0.307 | -4.287 | 0.181e-06^***^ |
| Animal (Red deer) | 0.073 | 0.297 | 0.249 | 0.803 |
| Animal (Roe deer) | -1.941 | 1.095 | -1.772 | 0.076 |
| Animal (Wild boar) | 0.060 | 0.287 | 0.210 | 0.833 |
| Site (Forest) | -0.043 | 0.517 | -0.085 | 0.932 |
| Season (Spring) | -1.047 | 0.524 | -1.998 | 0.045^.^ |
| Season (Summer) | 0.329 | 0.446 | 0.738 | 0.460 |
| Animal (Red deer): Site (Forest) | 0.072 | 0.448 | 0.161 | 0.872 |
| Animal (Roe deer): Site (Forest) | 2.271 | 1.110 | 2.046 | 0.040^.^ |
| Animal (Wild boar): Site (Forest) | -0.110 | 0.443 | -0.248 | 0.804 |
| Animal (Red deer): Season (Spring) | 0.943 | 0.564 | 1.670 | 0.094 |
| Animal (Roe deer): Season (Spring) | 1.800 | 0.703 | 2.560 | 0.010^*^ |
| Animal (Wild boar): Season (Spring) | 0.581 | 0.590 | 0.984 | 0.325 |
| Animal (Red deer): Season (Summer) | -0.848 | 0.484 | -1.752 | 0.079 |
| Animal (Roe deer): Season (Summer) | 0.151 | 0.589 | 0.257 | 0.797 |
| Animal (Wild boar): Season (Summer) | 0.254 | 0.491 | 0.519 | 0.603 |
| Significant codes: 0 ‘^***^’ 0.001 ‘^**^’ 0.01 ‘*’ 0.05 ^‘.’^ | | | | |
